# Social and Poor Law Problems

**Published:** 1907-01-19

**Authors:** 


### SOCIAL AND POOR LAW PROBLEMS.

### A WISE INEBRIATE.

A MONTH or two ago Richard Harris died in the workhouse of the Haslingden Union, after a residence there of about twelve years. At one time Harris was assistant postmaster and telegraphist at the Bacup Post Office, where his brother was postmaster. The brothers also owned the leading printing and stationery business in the town. Unfortunately Harris contracted bad habits, and became so reduced that he had to be removed to the workhouse. So far the tale is a sad but common one. The sequel, however, is better than that of most similar stories. In the workhouse it was soon found out that Harris was a capable as well as an educated man, and he was given the clerical work of the establishment to do. His ability was so great that the district auditor declared that nowhere else in his district were the books so well kept as they were by Harris at Haslingden. His friends more than once urged him to leave the workhouse and take a situation outside; but he invariably refused, on the ground that in the outer world he would be subject to the same temptations as formerly, and might return to his old habits. Only a month before his death the Guardians themselves offered to appoint him one of their officers; but this offer also he refused, because if he had accepted it he would have been compelled to live outside the workhouse, and again he feared temptation. Thus he remained to the end of his days nominally a pauper, though it is reckoned that his services saved the Guardians a salary of £3 a week. Doubtless he was wise in his choice; and he is to be respected for the discretion that made him remain in a safe though humble shelter. One would like to know, however, if the Guardians showed any special consideration for this ward of theirs who was to all intents and purposes a valuable servant. In similar cases, where the pauper earns more than his board, lodging, and clothing, it might be well to give special privileges, such as that of having meals apart from the mass of the inmates—who are for the most part the reverse of refined—or of sleeping elsewhere than in the ordinary dormitory. This would encourage a man who was more weak than wicked, and knew his own feebleness, to stay where he was protected against himself, and where the severe treatment which is necessary with many paupers would drive him out into the world again to his own destruction.

